# Titanium complex with an [OSSO]-type bis(phenolate) ligand for ethylene copolymerization with vinyl polar monomer based on group protection†

**DOI:** 10.1039/c9ra06271h

**Published:** 2019-08-27

**Authors:** Yuqiong Zhu, Sihan Li, Huaqing Liang, Xiuli Xie, Fangming Zhu

**Affiliations:** GDHPPC Lab, School of Chemistry and Chemical Engineering, Sun Yat-Sen University 510275 China ceszfm@mail.sysu.edu.cn +86-20-84114033 +86-20-84113250; Key Lab for Polymer Composite and Functional Materials of Ministry of Education, School of Chemistry and Chemical Engineering, Sun Yat-Sen University Guangzhou 510275 China

## Abstract

The [OSSO]-type bis(phenolate) titanium complex 1 activated by methylaluminoxane (MAO) was tested as a homogeneous catalyst for ethylene coordination copolymerization with protected vinyl polar monomer of *p-tert*-butyl-dimethylsilyloxystyrene (*p*-TBDMSOS). The results showed that the active species were almost not poisonous to the catalyst by the protected vinyl polar monomer. Moreover, the composition and sequence length as well as sequence distribution in the copolymers were investigated by theoretical calculation and ^13^C nuclear magnetic resonance (^13^C NMR) characterization. Especially, the incorporation ratio of *p*-TBDMSOS into the polyethylene chain could be controlled by changing *p*-TBDMSOS concentration in the feed. Interestingly, an approximate alternating copolymer of poly(*E-alt*-(*p*-TBDMSOS)) could be formed when the *p*-TBDMSOS feed concentration increased to 1.0 mol L^−1^. Subsequently, the poly(ethylene-*co*-(*p*-hydroxystyrene)) (poly(*E-co*-(*p*-HOS))) could be prepared by a facile deprotection in terms of desilylation of *tert*-butyldimethylsilyl ether. The hydrophilicity of poly(*E-co*-(*p*-HOS)) films were investigated by water contact angle measurements.

## Abstract 2

The [OSSO]-type bis(phenolate) titanium complex 1 activated by methylaluminoxane was used for the copolymerization of ethylene and protected vinyl polar monomer.

## Graphical abstract

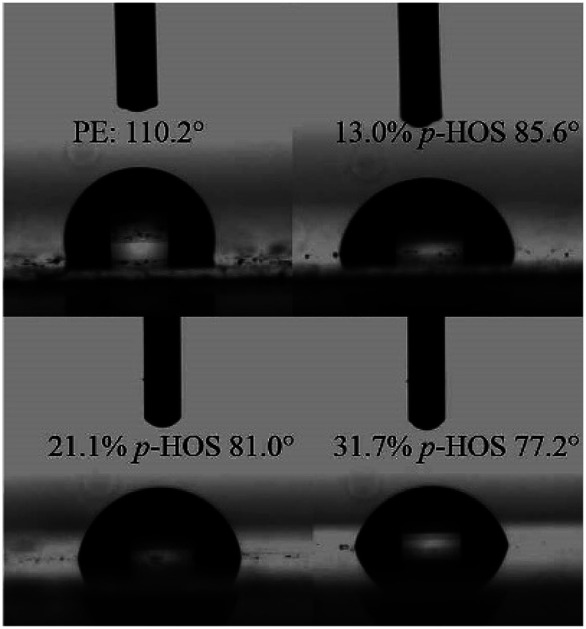

## Introduction

The introduction of even a small amount of vinyl polar monomer units into polyethylene (PE) backbone has attracted much attention because of the great improvement of its inherent properties, such as surface adhesivity, paintability and printability as well as blend miscibility with polar polymers.^1–3^ Accordingly, the synthesis of ethylene and vinyl polar monomer copolymers *via* coordination copolymerization has always been a very important and challenging topic.^4,5^ Since the middle of the 1990s, the late transition-metal catalysts corresponding nickel and palladium with sterically bulky ligands for coordination copolymerization of ethylene with vinyl polar monomers have been particularly concerned in the field of ethylene polymerization.^6–17^ Nevertheless, due to the poisoning effect of active species by polar group, the catalytic activity decreases significantly, the incorporation ratio remains at relatively low levels and the distribution of vinyl polar monomers in the main chain is almost random. In addition, highly branched, amorphous polymeric products were usually generated based on “chain walking”. Recently, ethylene copolymerization with vinyl polar monomers was also developed using rare-earth metal catalysts in terms of hetero-atom assisted olefins (such as anisyl propylenes) polymerization (HOP) mechanism, as reported by Hou and Cui *et al.*^18–23^ As a consequence, polymers of polyethylene containing vinyl polar monomer units were prepared with controllable molecular weight and incorporation ratio.

Especially, it is considerably difficult to accomplish directly coordination polymerization of vinyl polar monomers promoted by titanium-based catalysts in consequence of the poisoning effect of active species by polar group. Consequently, these vinyl polar monomers were generally protected using bulky groups before polymerization.^24–32^ As reported by Kawabe *et al.*^33,34^ and Kim *et al.*,^35^ when the bulky *tert*-butyl(dimethyl)silyl protective group was used, the isotactic or syndiotactic poly(*p*-hydroxystyrene) (poly(*p*-HOS)) as well as styrene-based copolymers could be synthesized based on coordination polymerization with titanium-based complexes and methylaluminoxane (MAO). To best of our knowledge, the copolymerization of ethylene with vinyl polar monomer or protected vinyl polar monomer is less reported with titanium-based catalysts.^24,36^ Wu and his co-workers exhibited copolymerization of ethylene with 10-undecen-1-ol, 10-undecenoic acid, and 5-hexen-1-ol using triisobutylaluminum as a protection reagent catalyzed by phenoxy-imine (FI) titanium catalysts. Nevertheless, the incorporation ratio of polymers was less than 7 wt%.^36^ In this contribution, we demonstrated ethylene copolymerization with vinyl polar monomer on the basis of the strategy of group protection catalyzed by 1,4-dithiabutandiyl-2,2′-bis(6-cumenyl-4-methylphenoxy) titanium dichloride^37^ (complex 1) in the presence of MAO. Consequently, the poly(*E-co*-(*p*-HOS)) with controllable incorporation ratio of *p*-HOS into polyethylene chains by changing concentrations in the feed could be prepared by shielding hydroxyl group with *tert*-butyldimethylsilyl ether as a comonomer (*p-tert*-butyldimethylsilyloxystyrene (*p*-TBDMSOS)) used for ethylene coordination copolymerization and subsequent deprotection to form *p*-HOS units.^38,39^ Note that the introduction of *p*-HOS units could tremendously improve the surface hydrophilicity of the resultant copolymer films.

## Results and discussion

**Scheme 1:**
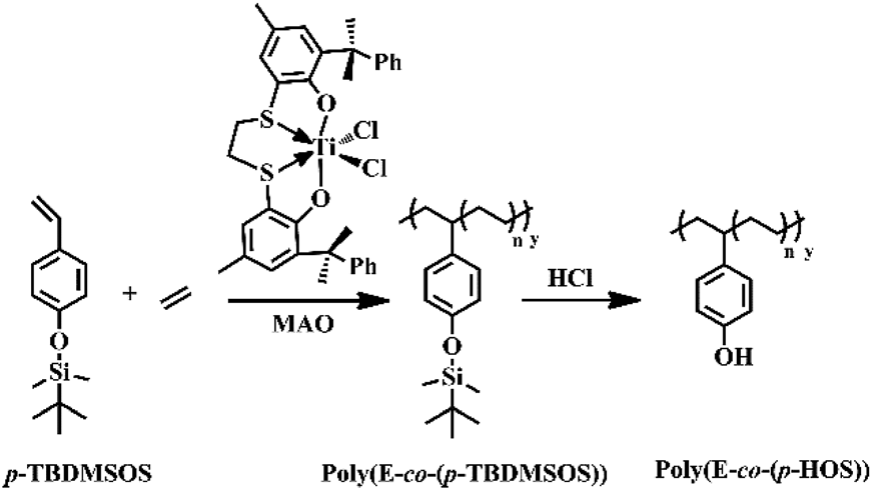
The synthesis routes for poly(ethylene-*co*-(*p*-TBDMSOS)) and deprotection of hydroxyl groups to form poly(ethylene-*co*-(*p*-HOS)) catalyzed by complex 1 and MAO.

As reported by Okuda *et al.*,^40,41^ 5-5-5 [OSSO]-type bis(phenolate) titanium complexes activated by MAO could promote ethylene-styrene copolymerization with high catalytic activity. Moreover, by using the same catalyst system, Proto and his coworkers accomplished the copolymerization of ethylene and some *para*-substituted styrene (*p*-methylstyrene, *p-tert*-butylstyrene, *p*-bromostyrene and *p*-chlorostyrene).^42^ In this case, we demonstrated the copolymerization of ethylene with *t*-butyldimethylsilyl-protected *p*-HOS (*p*-TBDMSOS) catalyzed by complex 1 upon activation with MAO and the sequential deprotection of hydroxyl groups by hydrochloric acid to afford a linear and hydrophilic polymer of poly(*E-co*-(*p*-HOS)) (Scheme 1).

Under 1.2 atm of ethylene pressure, the influences of different *p*-TBDMSOS concentrations in toluene in the feed on the copolymerization behaviours, structure parameters and thermal properties of the resultant copolymers were summarized in Table 1. To avoid the formation of PE homopolymer after *p*-TBDMSOS was entirely consumed at low *p*-TBDMSOS feed concentrations, the copolymerization reactions were stopped after 3 min.

**Table 1:** Copolymerization of ethylene with *p*-TBDMSOS catalyzed by complex 1 in the presence of MAO^a^

Run	*p*-TBDMSOS (mol L^−1^)	Yield (g)	Activity b	TOF × 10^−4^^c^	*M*_w_ d × 10^−4^		*M*_w_/*M*_n_^d^	*T*_m_ or *T*_g_^e^ (°C)	Conv. (%)	Incrop.^f^ (mol%)
				*E*	*p*-TBDMSOS					
1	0.00	0.15	1.5	5.36	—	0.86	1.69	118.1	—	0
2	0.039	0.30	3.0	6.00	0.73	1.23	1.41	−25.9, 5.9	83.4	10.9
3	0.058	0.38	3.8	6.09	0.91	1.24	1.51	−19.4, 19.0	77.7	13.0
4	0.078	0.43	4.3	5.92	1.24	1.30	1.54	−13.5, 28.2	74.8	17.3
5	0.10	0.56	5.6	6.20	1.66	1.39	1.54	−9.9, 38.4	82.4	21.1
6	0.20	0.96	9.6	7.05	3.27	2.43	1.84	7.1	80.9	31.7
7	1.0	2.61	26.1	9.90	10.3	3.92	2.37	43.4	49.7	50.9

a Polymerization conditions: titanium complex 1, 2.0 μmol; ethylene pressure, 1.2 atm; polymerization time, 3 min; toluene as solvent, total volume = 20 mL; Al/Ti = 1200; polymerization temperature, 30 °C.

b Catalyst activity in 10^6^ g (polymer) (mol Ti)^−1^ h^−1^.

c TOF = mol of polymer consumed per mol catalyst per h (mol P mol^−1^ Ti h^−1^).

d Determined by GPC in 1,2,4-trichlorobenzene (TCB) at 150 °C and in THF at 40 °C with polystyrene standards.

e Determined by DSC.

f Determined by ^1^H NMR.

The complex 1/MAO presented a good catalyst system for ethylene homopolymerization and copolymerization with *p*-TBDMSOS. The catalytic activity gradually increased with the increase of the *p*-TBDMSOS concentrations in the feed resulting from the much higher molecular weight of *p*-TBDMSOS unit than that of ethylene unit. Moreover, the polymerization activity of monomers was also expressed as turnover frequency (TOF). The TOF of ethylene was nearly uninfluenced while the TOF of *p*-TBDMSOS increased by changing the *p*-TBDMSOS feed concentrations ranging from 0.039 to 1.0 mol L^−1^ at 30 °C, revealing that active species were almost not poisoned by the protected vinyl polar monomer. Consequently, the catalyst exhibited good catalytic behaviour for copolymerization of *p*-TBDMSOS with ethylene.

**Fig. 1:**
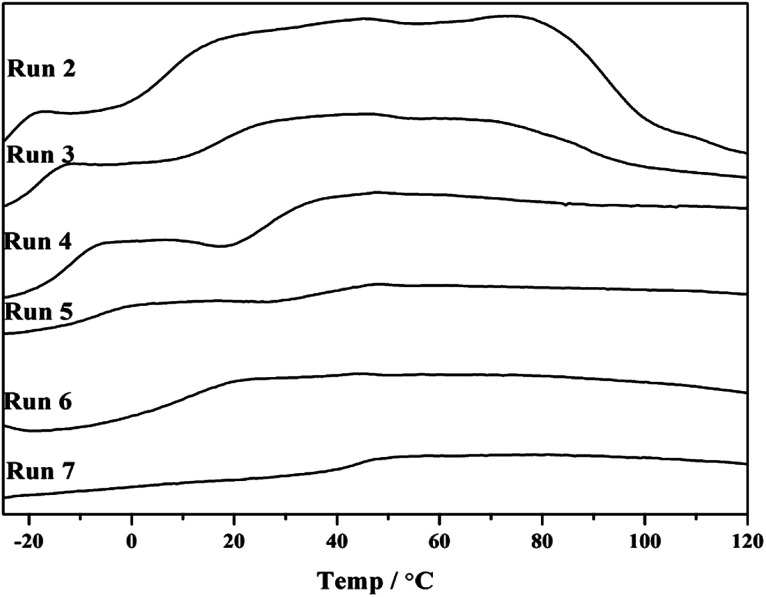
DSC profiles of copolymerization products obtained from Runs 2–7 in Table 1.

Note that *p*-TBDMSOS concentration gradually decreased as *p*-TBDMSOS was continuously consumed during the copolymerization process. Accordingly, the copolymerization products displayed two glass transition temperature (*T*_g_) when the *p*-TBDMSOS feed concentration was less than 0.20 mol L^−1^ (Runs 2–5 in Fig. 1), indicating that they mainly consisted of two parts. The copolymer with higher *T*_g_ presents higher fraction of *p*-TBDMSOS units at the early stage, and the copolymer with lower *T*_g_ indicates that the sequence lengths of ethylene increased at the late stage. It is a remarkable fact that only a single *T*_g_ at higher *p*-TBDMSOS feed concentrations of Runs 6 and 7 were observed respectively (Fig. 1), which is probable that the concentration of *p*-TBDMSOS could maintain a relatively high value during the copolymerization process.

**Fig. 2:**
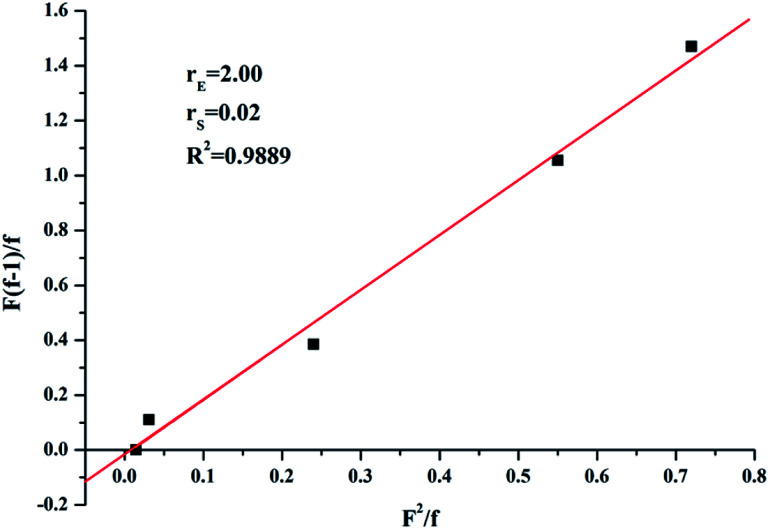
Fineman–Ross plot for copolymerization of ethylene with *p*-TBDMSOS using complex 1 and MAO.

In order to analysis the distribution of ethylene units and *p*-TBDMSOS units in the copolymer chains, the reactivity ratios of ethylene (*r*_E_) and *p*-TBDMSOS (*r*_*p*-TBDMSOS_) were calculated by means of the Fineman–Ross equation.
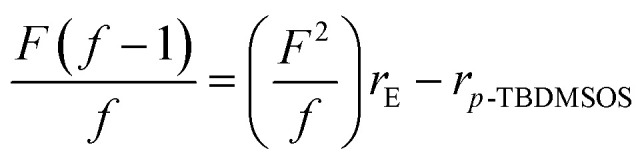
where *F* is the feeding monomer composition ratio and *f* is the copolymer composition ratio which can be determined by ^1^H NMR analysis. Fig. 2 shows a plot of 
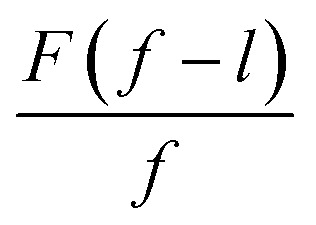
*versus*
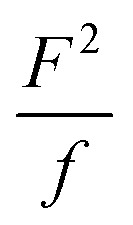
 for the copolymerization of ethylene with *p*-TBDMSOS, revealing a straight line (*R*^2^ = 0.9889). The slope and intercept of the line are equal to *r*_E_ = 2.0 and *r*_*p*-TBDMSOS_ = 0.02, respectively, which are close to those of ethylene and styrene calculated by Okuda and his coworker (*r*_E_ = 1.2, *r*_S_ = 0.031).^41^ The result also indicates that the group of *tert*-butyldimethylsilyl ether has little influence on the rate of copolymerization. Moreover, the *r*_E_ value (>1) indicates a preference for the insertion of ethylene whatever the last inserted monomer unit is in each copolymerization.

**Scheme 2:**
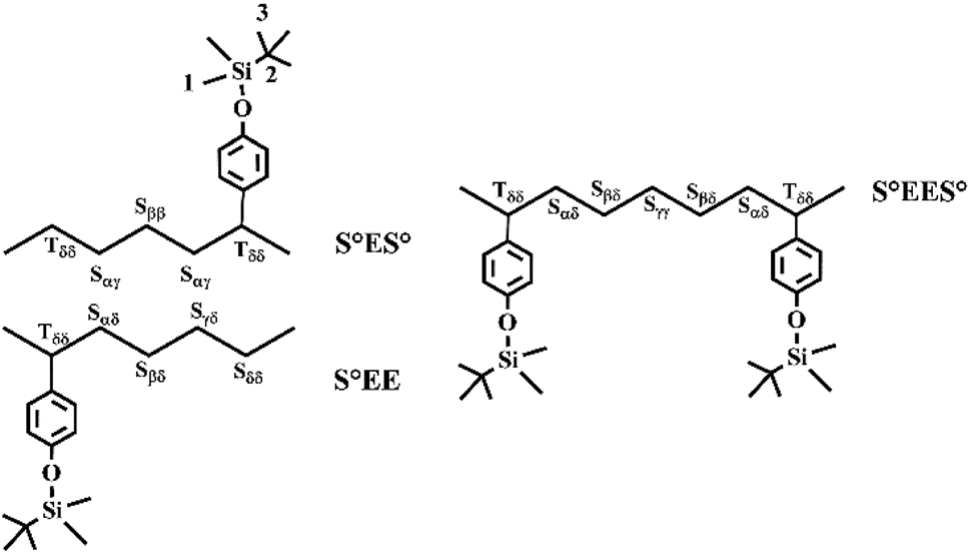
Monomer sequences in poly(*E-co*-(*p*-TBDMSOS)).

**Fig. 3:**
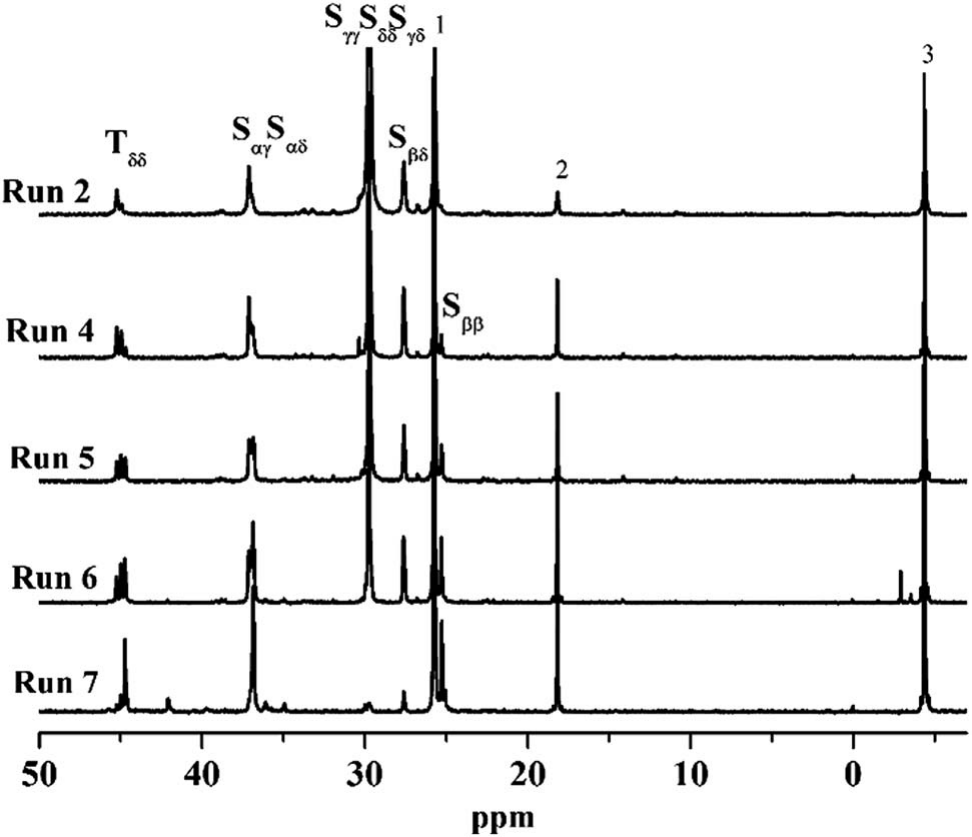
Aliphatic regions of ^13^C NMR spectra of poly(*E-co*-(*p*-TBDMSOS)) with different incorporation ratios of *p*-TBDMSOS 10.9 mol% (Table 1, Run 2), 17.3 mol% (Run 4), 21.1 mol% (Run 5), 31.7 mol% (Run 6) and 50.9 mol% (Run 7).

The carbon terminology follows that of Carman and Wilkes,^43^ where S and T refer to the secondary (methylene) and tertiary (methine) carbons of the main chain, respectively. The position of carbon atom relative to its nearest T groups was labeled by two Greek subscripts where *δ* indicates all T carbons four or farther than four bonds away from the S carbon as shown in Scheme 2. Fig. 3 shows the aliphatic regions of ^13^C NMR spectra of poly(*E-co*-(*p*-TBDMSOS)) with different incorporation ratios of *p*-TBDMSOS. The absence of signal *δ* 42.0 ppm attributed to T_αα_ of the S°S° (S° = *p*-TBDMSOS) sequences suggesting that the ethylene sequences in the copolymer are separated by isolated *p*-TBDMSOS units at lower comonomer feed concentrations (Runs 2–6). Moreover, the ethylene sequence lengths are shortened as evidenced by the signal at *δ* 30 ppm, attributed to the presence of long ethylene sequences (EEE), dropping distinctly with the increase of the initial *p*-TBDMSOS feed concentrations (Runs 5–7).^44^

In addition, let *P*_EE_ be the probability that a growing chain active species (E*) will add to monomer ethylene (M_E_). To a good approximation the only two possible fates of E* are addition of M_E_ or addition of monomer *p*-TBDMSOS (M_*p*-TBDMSOS_). Hence, it is possible to write this probability as
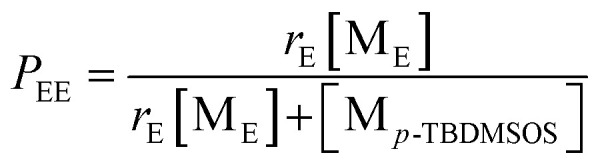


Therefore, in consideration of the relationship between *P*_EE_ and *r*_E_, the average sequence lengths of ethylene units (*l*_E_) can be calculated as
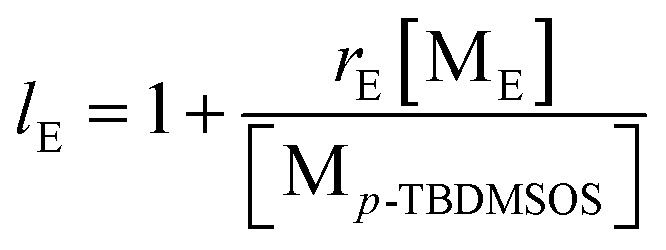


Similarly, the average sequence lengths of *p*-TBDMSOS units (*l*_*p*-TBDMSOS_) is given by
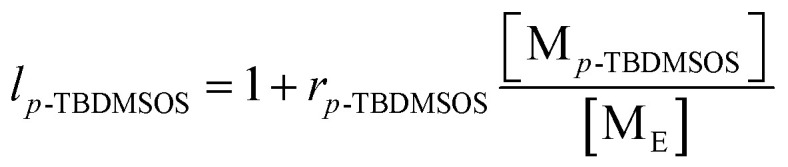


The value of *l*_E_ increases, while the value of *l*_*p*-TBDMSOS_ decreases as result of the constant ethylene pressure in combination with the continuous reduction of *p*-TBDMSOS concentration during copolymerization process. Furthermore, the calculation results indicate that even though the value of *l*_*p*-TBDMSOS_ is approaching to 1 whereas the value of *l*_E_ is much larger than 2 in Runs 2–6, which in accordance with the observation of ^13^C NMR that the ethylene sequences are separated by isolated *p*-TBDMSOS units at lower *p*-TBDMSOS feed concentrations. On the other hand, 49.7% of *p*-TBDMSOS was consumed as result of copolymerization for 3 min (Run 7), corresponding to a *p*-TBDMSOS concentration ranging from 1.0 to 0.5 mol L^−1^. Therefore, the values of *l*_E_ and *l*_*p*-TBDMSOS_ were changed from 1.3 to 1.5 and 1.1 to 1.0, respectively, indicating the formation of approximate alternating copolymer of poly(*E-alt*-(*p*-TBDMSOS)) with a *T*_g_ of 43.4 °C. As a consequence, there is a strong tendency of the complex 1/MAO catalyst to produce alternating ES°E (E = ethylene, S° = *p*-TBDMSOS) sequences in the copolymers with comonomer concentration as evidenced by the presence of the S_ββ_ methylene carbon relative to the alternating sequences in the Run 7 with *p*-TBDMSOS feed concentration of 1.0 mol L^−1^.

**Fig. 4:**
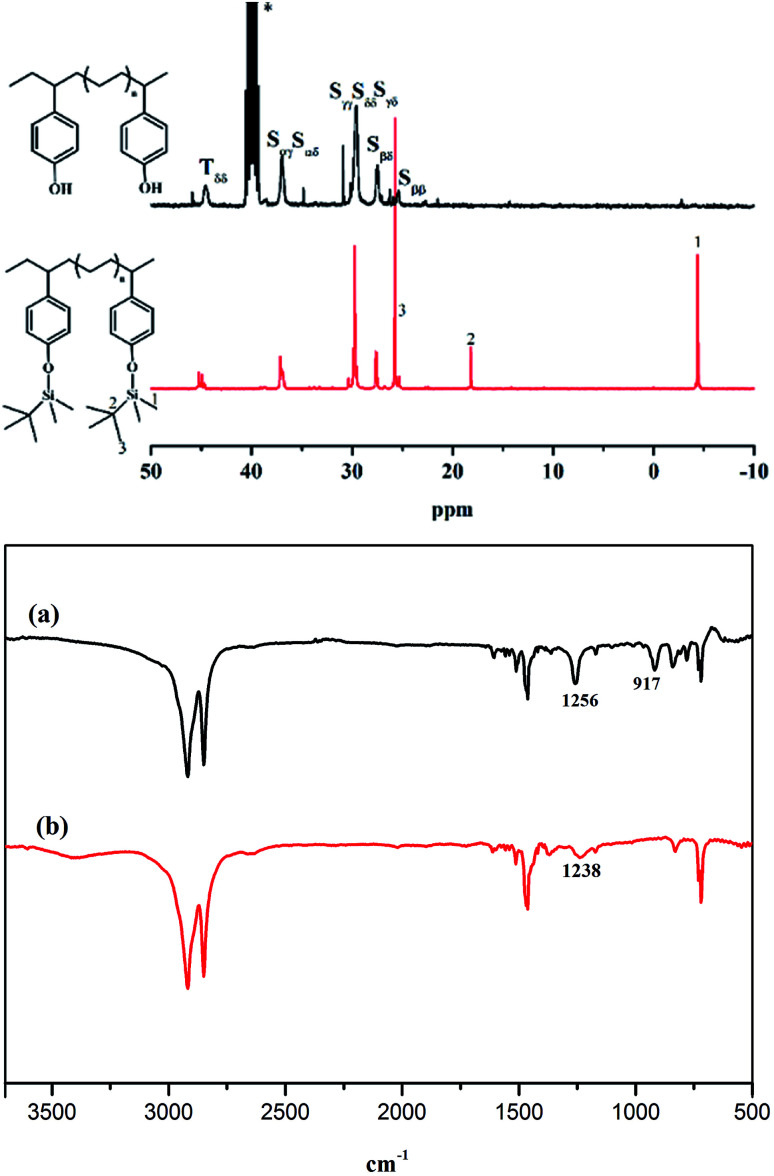
The ^13^C NMR spectra of poly(*E-co*-(*p*-TBDMSOS)) (a) in CDCl_3_ and poly(*E-co*-(*p*-HOS)) (b) in DMSO-*d*_6_ and FT-IR for Run 5 in Table 1.

Poly(*E-co*-(*p*-TBDMSOS)) was easily deprotected by desilylation based on acidification so as to from poly(*E-co*-(*p*-HOS)).^45^Fig. 4 displays the typical ^13^C NMR spectra of poly(*E-co*-(*p*-TBDMSOS)) from Run 5 in Table 1 and poly(*E-co*-(*p*-HOS)) in terms of desilylation. The absence of characteristic peaks at −4.55, 17.99 and 25.63 ppm attributed to the *tert*-butyldimethylsilyl group after deprotection. We further confirmed the resulting polymers by IR spectra. As mentioned in Fig. 4a, the high peak at 1257 cm^−1^ and 917 cm^−1^ were corresponding to the symmetric deformation vibration of methyl of Si–CH_3_ and the stretching vibration of Si–C, respectively. The disappear of these peaks in Fig. 4b and the forming of a new peak at 1238 cm^−1^ attributed to the C–O stretching vibration of phenol also confirmed the success of deprotection and the formation of P(*E-co*-(*p*-HOS)), which was in line with the NMR analyses.

**Fig. 5:**
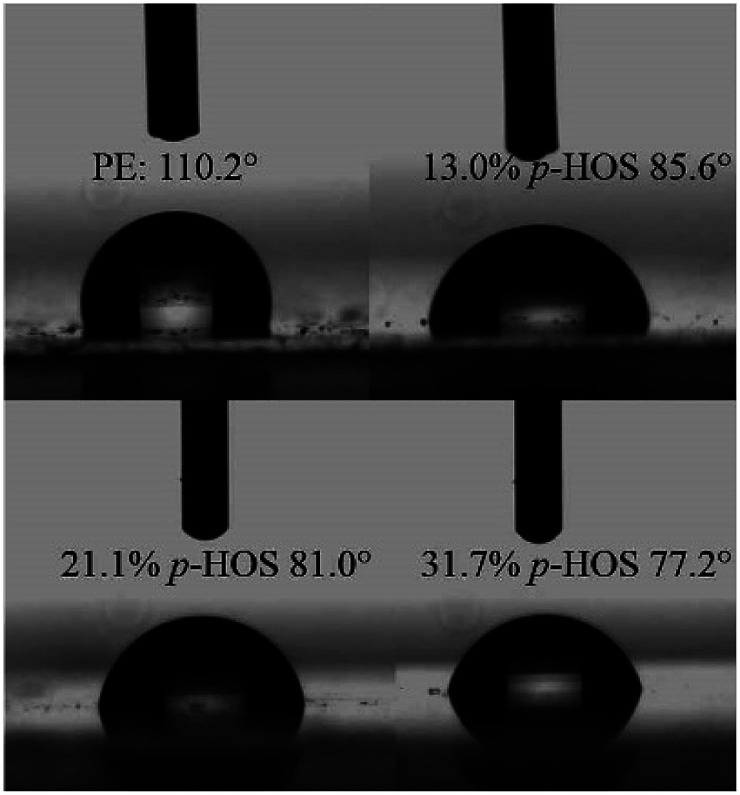
The photographs of water contact angle on polyethylene films and poly(*E-co*-(*p*-HOS)) films containing different incorporation ratios of *p*-HOS of 13.0 mol%, 21.1 mol% and 31.7 mol% at 25 °C.

Note that the introduction of *p*-HOS into polyethylene back-bone could dramatically improve its hydrophilicity.^46^Fig. 5 displays the representative photographs of a water droplet on polyethylene and poly(*E-co*-(*p*-HOS)) films at 25 °C. The water contact angle (*θ*) on polyethylene film is about 110°, indicating polyethylene is a hydrophobic material. Nevertheless, when the incorporation ratio was 13.0 mol%, the WCA exhibited a rapid decline to 85.6°. Furthermore, the contact angle decreases strikingly with increasing the incorporation of *p*-HOS units in poly(*E-co*-(*p*-HOS)).

## Experimental

### Materials

Toluene and hexane were refluxed over sodium or potassium using benzophenone as a moisture indicator under nitrogen atmosphere. MAO solution (1.5 M in toluene) was purchased from Akzo-Nobel and was used as received. Styrene was dried with calcium hydride at room temperature for about 24 h and distilled under vacuum. Titanium tetrachloride solution (1.0 M in toluene) and *n*-butyllithium solution (1.0 M in hexane) were purchased from J & K Scientific. Other reagents, if not specified, were purchased from Aladdin and J & K Scientific and used as received. Complex 1 and *p*-TBDMSOS were synthesized according to the procedure reported by previous literature.^37,47,48^

### Copolymerization of ethylene with *p*-TBDMSOS

A 100 mL Schlenk flask equipped with a magnetic stirrer was dried more than 3 h by infrared light under vacuum and then cooled to room temperature. After replacement with high-pure nitrogen three times and ethylene twice to atmospheric pressure, toluene, MAO and *p*-TBDMSOS were introduced into the ethylene-filled reactor. Copolymerization was initiated by injecting a toluene solution of the complex 1 into the reactor. After copolymerization for 3 min, 4 mL methanol was added to terminate the reaction and then the resultant mixture was added into acidic ethanol (10 mL 37 wt% hydrochloric acid/100 mL ethanol) to wash and purify. The copolymers of poly(*E-co*-(*p*-TBDMSOS)) was isolated by filtration, washed with ethanol several times, and dried to constant weight under vacuum at 40 °C.

### Deprotection of poly(*E-co*-(*p*-TBDMSOS))

400 mg poly(*E-co*-(*p*-TBDMSOS)) was thoroughly dissolved in tetrahydrofuran (THF) and acidified with 8 mL 37 wt% hydrochloric acid. Subsequently, the mixture was refluxed for 6 h to remove *tert*-butyldimethylsilyl group and generate poly(*E-co*-(*p*-HOS)). Then the resultant copolymer was centrifuged, washed with ethanol and dried to a constant weight under vacuum.

### Measurements

Molecular weight and molecular weight distribution (*M*_w_/*M*_n_) of polymers were measured by high temperature gel permeation chromatography (HT-GPC) calibrated by narrow molecular weight distribution polystyrene standards using a Varian PL-220 HTGPC equipped with a triple-detection detectors consisting of a two-angle (15 and 90°) light scattering (LS) detector, a differential refractive-index detector and a four-bridge capillary viscometer at 150 °C with 1,2,4-trichlorobenzene as solvent. The normal temperature GPC was examined on a Waters (2414 refractive index detector) at 40 °C with THF as solvent. ^1^H NMR (400 MHz) and ^13^C NMR (400 MHz) spectra of polymers were recorded in a Varian Unity Inova 400 spectrometer in CDCl_3_ or DMSO-*d*_6_. Differential scanning calorimetry (DSC) experiments were carried out on PerkinElmer DSC-4000 instrument at 10 °C min^−1^ over the temperature ranging from −50 to 300 °C for standard measurements. Water contact angles on the polymer films were carried out on DSA100 by the dynamic sessile drop method. Polymer films were prepared by spin coating of 3 to 5% (w/w) solution in dimethyl formamide (DMF) onto glass. The solution was dried off the glass by heating for a few hours, and then a second layer was applied and dried. The water contact angles were measured at 25 °C and the values were the average of at least 6 measurements.

## Conclusions

Coordination copolymerization of ethylene with protected polar vinyl monomer of *p*-TBDMSOS is performed catalyzed by complex 1 upon activation with MAO, which indicates that the polar groups could prevent the deactivation caused by polar monomers with the protection of *tert*-butyldimethylsilyl ether. Copolymerization parameters are calculated to be *r*_E_ = 2.0 for ethylene and *r*_*p*-TBDMSOS_ = 0.02 for *p*-TBDMSOS (*r*_E_*r*_*p*-TBDMSOS_ = 0.04), tending to from alternating copolymers. Consequently, an approximate poly(*E-alt*-(*p*-TBDMSOS)) can be formed at 1.0 mol L^−1^*p*-TBDMSOS in the feed. Most interestingly, the molar incorporation ratio of *p*-TBDMSOS into polyethylene chain can be controllable by adjusting *p*-TBDMSOS feed concentration in each copolymerization. Note that the ethylene sequences are separated by isolated *p*-TBDMSOS units at lower *p*-TBDMSOS feed concentrations. Moreover, a facile deprotection of *p*-TBDMSOS units gives rise to the formation of polar monomer units of *p*-HOS in polyethylene chain as a result of the desilylation of *tert*-butyldimethylsilyl ether with hydrochloric acid. More remarkably, the introduction of *p*-HOS into polyethylene backbone can dramatically improve its hydrophilicity.

## Conflicts of interest

There are no conflicts to declare.

## Supplementary Material

RA-009-C9RA06271H-s001
