# Supplementary material for: Titanium complex with an [OSSO]-type bis(phenolate) ligand for ethylene copolymerization with vinyl polar monomer based on group protection
Source: RSC Adv. 2019 Aug 27;9(46):26582–7. doi: 10.1039/c9ra06271h (PMC9070498; doi:10.1039/c9ra06271h)
Supplement: RA-009-C9RA06271H-s001 [file RA-009-C9RA06271H-s001.pdf]

Electronic Supplementary Material (ESI) for RSC Advances.
This journal is © The Royal Society of Chemistry 2019

Supplementary Materials for:

Titanium complex with an [OSSO]-type bis(phenolate) ligand for ethylene copolymerization with vinyl polar monomer

Yuqiong Zhu[1], Sihan Li[1], Huaqing Liang[1], Xiuli Xie[1], Fangming Zhu[1, 2]

[1]GDHPPCLab, School of Chemistry, Sun Yat–Sen University, 510275, China

[2]Key Lab for Polymer Composite and Functional Materials of Ministry of Education, School of Chemistry, Sun Yat-Sen University, Guangzhou, 510275, China

Contents

1. Experimental Procedures……………………………………………………..S2

2. Spectra Data…………………………………………………………………...S2

2.1 ^{1}H NMR and ^{13}C NMR……………………………………………………..S3

2.2 DSC data………………………………………………………………………S3

3.References……………………………………………………………………...S4

Experimental Procedures

Synthesis of *p*-TBDMSOS. A 500 mL flask equipped with a magnetic stirrer was charged with 14.46 g (120 mmol) of p-hydroxybenzaldehyde and 100 mL of CH_2Cl_2. Then 16.8 mL (120 mmol) of trimethylamine was introduced into the suspension solution and stirred until it become clear. Then 100 mL solution of CH_2Cl_2 containing 18.04 g (120 mmol) *tert*-butyldimethylsily was added dropwise into the solution and stirred 2 h at room temperature. The reactant was quenched by adding 100 mL water. The organic phase was separated and washed three times by pure water and brine respectively, dried by anhydrous $MgSO_4$, filtered and concentrated to yield yellow oily production, which was regard as *p*-*tert*-butyldimethylsilyxybenzaldehyde. Yield 26.8 g (95%).[1]

THF (50 mL) was added to dissolve methyltriphenylphosphonium bromide (10.18 g, 28.5 mmol) in a 250 mL flask equipped with a magnetic stirrer. *n*-BuLi (2.5 M solution in hexane, 11.2 mL) in 50 mL THF was introduced dropwise via syringe into the solution above under ice bathe and the mixture was stirred 10 min. A solution of *p*-*tert*-butyldimethylsilyxybenzaldehyde (7.09 g, 30 mmol) in 50 mL THF was added dropwise with a dropping funnel and then stirred for 4 h after heating up to the room temperature. After the reaction, the reactant was quenched with NH_4Cl (1.6 g) and filtered to obtain the solution. Then it was purified by concentrating and column chromatography with hexane as the eluent. The final production was a colorless oil with high yield of 5.63 g (90 %).

Spectra Data

^{1}H NMR and ^{13}C NMR

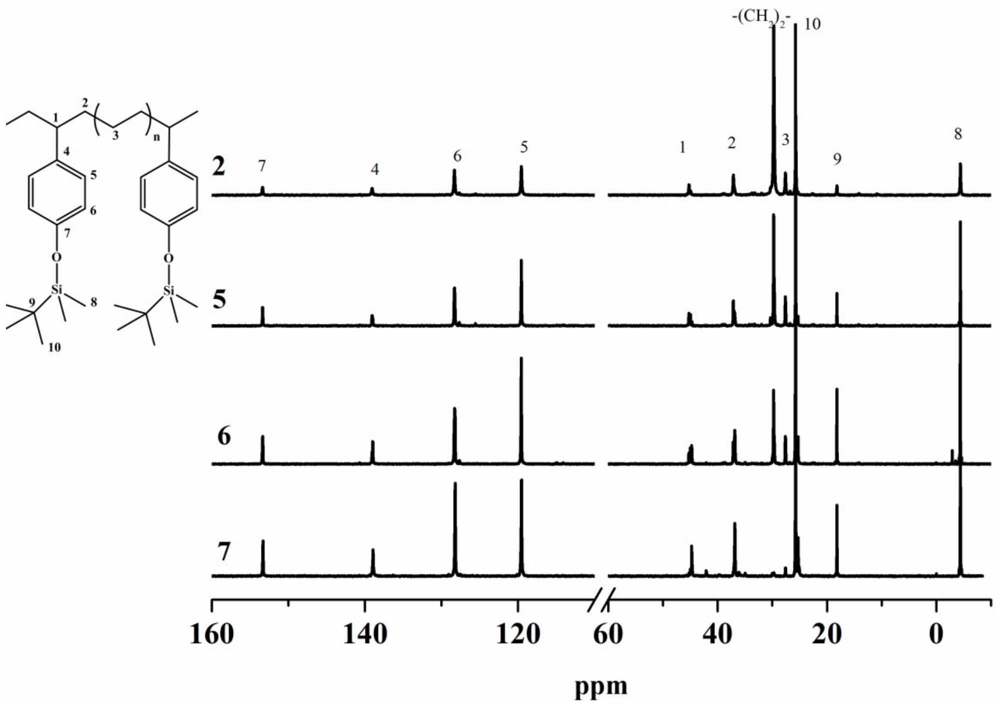

Fig. 1. The ^{13}C NMR spectra of poly(E-*co*-(*p*-TBDMSOS)) obtained from Runs 2, 5, 6 and 7 in Table 1.

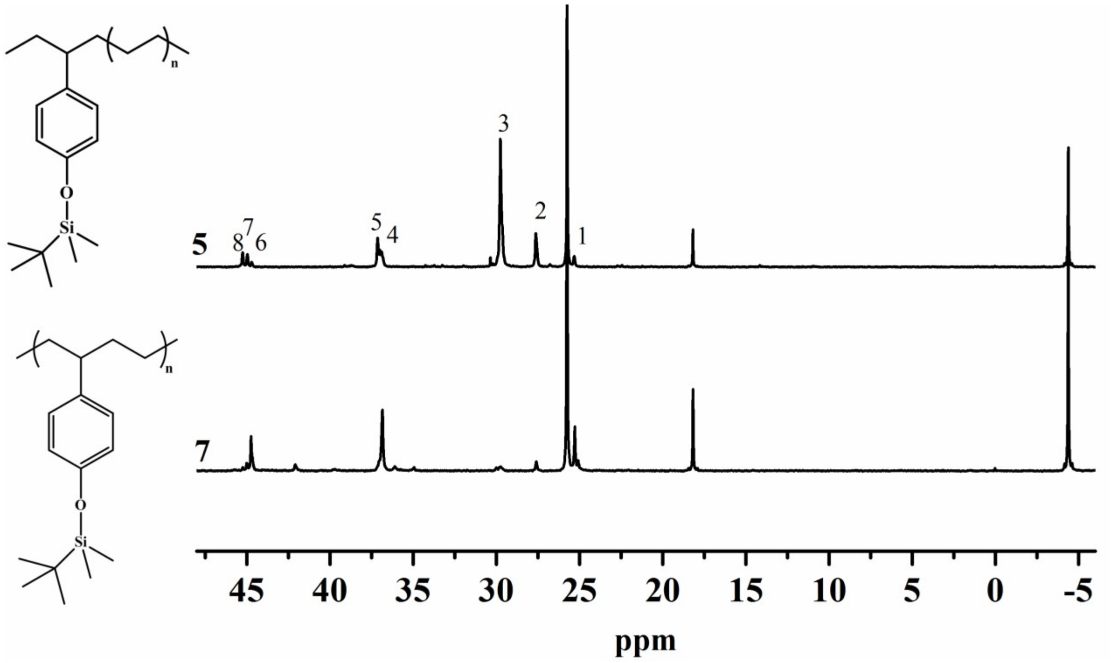

Fig. 2. Aliphatic regions of ^{13}C NMR spectra of poly(E-*co*-(*p*-TBDMSOS))

Table 1. The ^{13}C NMR chemical shift of poly(E-*co*-(*p*-TBDMSOS)) in Fig. 2

Carbon	Chemical Shift (ppm)	Sequence
1($S_{\beta\beta}$)	25.3	SES
2($S_{\beta\delta}$)	27.6	SEES, SEE
3($S_{\gamma\gamma}$, $S_{\delta\delta}$, $S_{\gamma\delta}$)	29.7	SEES, EEE, SEE
4($S_{\alpha\gamma}$)	36.9	SES
5($S_{\alpha\delta}$)	37.1	SEES
6($T_{\delta\delta}$)	44.7	SES
7($T_{\delta\delta}$)	45.0	SEES
8($T_{\delta\delta}$)	45.2	SEE

DSC and GPC data

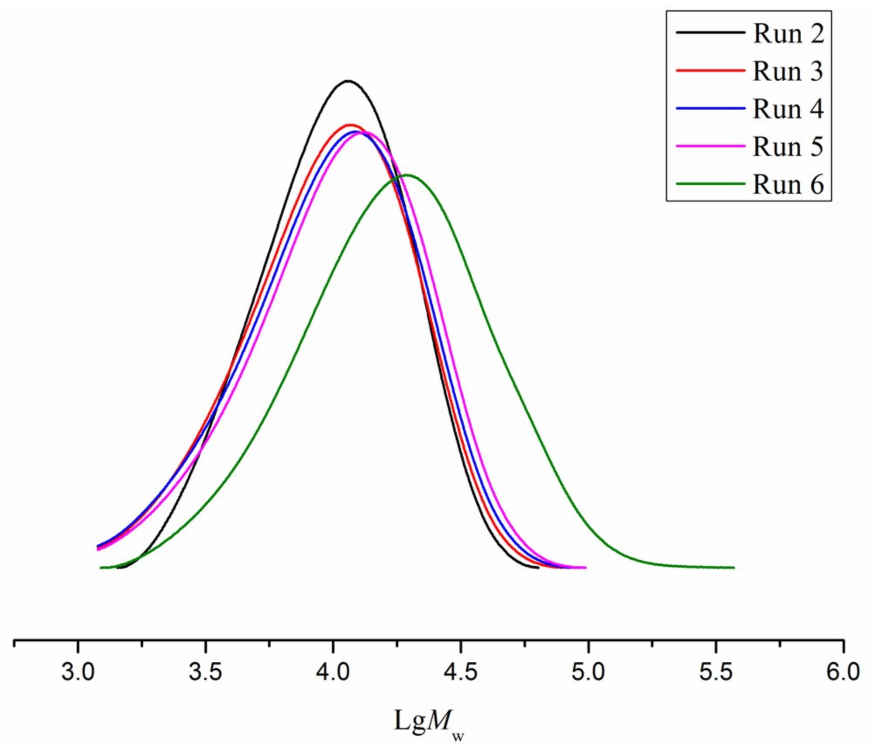

Fig. 3. GPC curves for copolymerization of E/*p*-TBDMSOS of Runs 2, 3, 4, 5 and 6.

3. References

(1) Kim, Y.; Do, Y. Preparation of Syndiotactic Poly(4-*tert*-butyldimethylsilyloxystyrene) and

Poly(4-hydroxystyrene). *Macromol. Rapid Commun.* **2000**, 21, 1148–1155.
